# From coarse to fine: Two-stage deep residual attention generative adversarial network for repair of iris textures obscured by eyelids and eyelashes

**DOI:** 10.1016/j.isci.2023.107169

**Published:** 2023-06-21

**Authors:** Ying Chen, Yugang Zeng, Liang Xu, Shubin Guo, Ali Asghar Heidari, Huiling Chen, Yudong Zhang

**Affiliations:** 1 School of Software, Nanchang Hangkong University, Nanchang, Jiangxi 330063, China; 2 School of Surveying and Geospatial Engineering, College of Engineering, University of Tehran, Tehran, Iran; 3 Key Laboratory of Intelligent Informatics for Safety & Emergency of Zhejiang Province, Wenzhou University, Wenzhou 325035, PR China; 4 School of Computing and Mathematical Sciences, University of Leicester, LE1 7RH Leicester, UK

**Keywords:** Health sciences, Medicine, Optometry

## Abstract

We propose a two-stage deep residual attention generative adversarial network (TSDRA-GAN) for inpainting iris textures obscured by eyelids. This two-stage generation approach ensures that the semantic and texture information of the generated images is preserved. In the second stage of the fine network, a modified residual block (MRB) is used to further extract features and mitigate the performance degradation caused by the deepening of the network, thus following the concept of using a residual structure as a component of the encoder. In addition, for the skip connection part of this phase, we propose a dual-attention computing connection (DACC) to computationally fuse the features of the encoder and decoder in both directions to achieve more effective information fusion for iris inpainting tasks. Under completely fair and equal experimental conditions, it is shown that the method presented in this paper can effectively restore original iris images and improve recognition accuracy.

## Highlights

• A two-stage deep residual attention generation adversarial network is proposed
• The two-stage generation approach ensures the semantic and texture information
• DACC is proposed to learn the location information of the region to be repaired
• MRB is proposed to further extract features

## Graphical abstract

**Figure:**
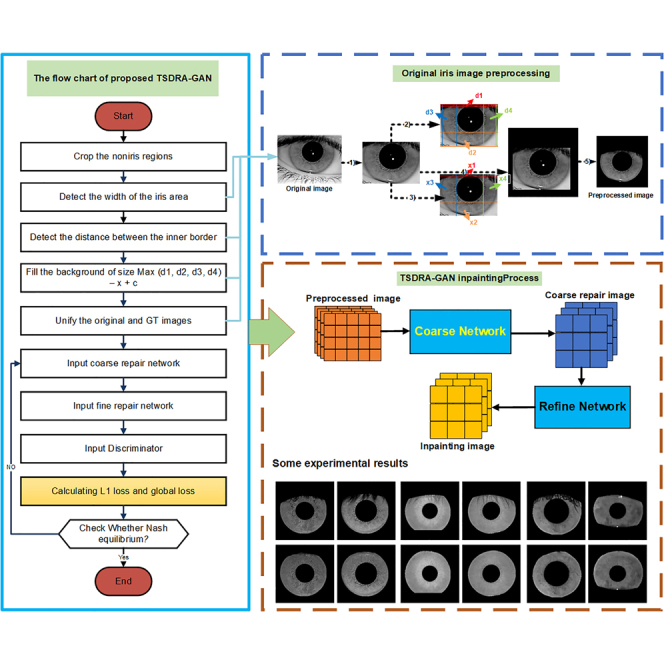

## Introduction

**Figure 1:**
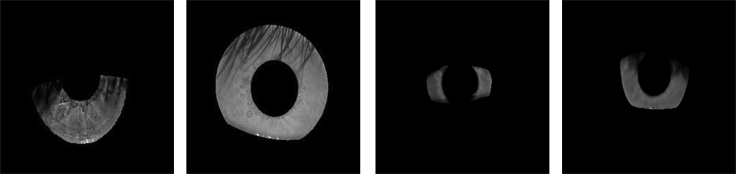
Several common low-quality iris images

Since Daugmann's groundbreaking work in 1993,^1^ significant progress has been made in the field of iris recognition using near-infrared imaging in a controlled setting.^2^ In order for this form of diagnosis to be successful, it is essential for patients to adhere to the rules on how to properly capture iris images, such as standing in the right position and maintaining an eye contact with the camera without squinting.^3^ Iris recognition systems are currently employed in a multitude of settings, such as international border control, access security to banking ATMs, electronic passports for biometric authentication, forensic investigation, smartphone authentication, computer logins, and car theft mitigation technologies.^4^^,^^5^^,^^6^ Most of the iris images in these scenes are acquired from subjects in a noncooperative state. Under uncontrolled circumstances or without the cooperation of the individual whose iris is being depicted, the clarity and precision of iris images is often diminished due to the introduction of noise such as motion blur, specular reflection, and hazy quality,^7^ eyelashes and eyelid shields, off-angle views, and other artifacts, thus ultimately deteriorating recognition performance. Figure 1 shows several common low-quality iris images affected by eyelash and eyelid occlusion and noncooperation.

**Figure 2:**
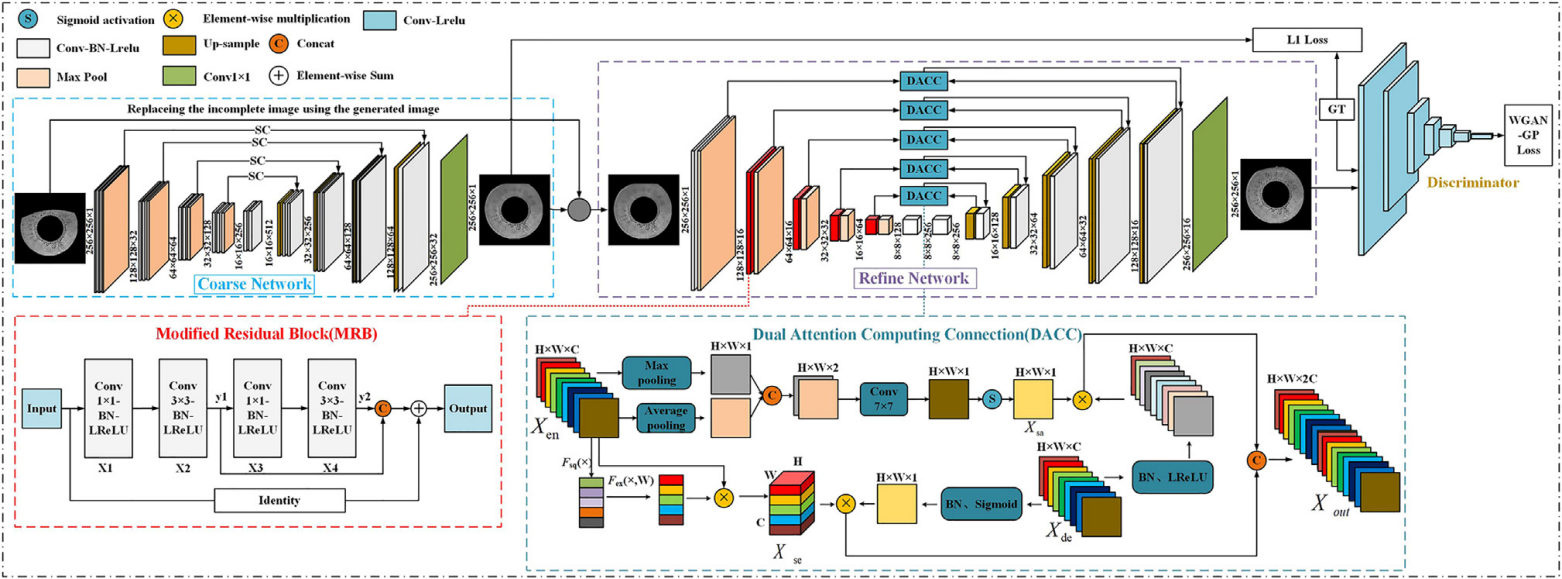
TSDRA-GAN structure diagram

To address the above issues, a deep-learning-based and end-to-end method of repairing obscured iris textures to enrich iris texture information and improve iris recognition accuracy is proposed in this paper, structure diagram of TSDRA-GAN is shown in Figure 2. Image inpainting methods can be broadly classified into nonsemantic and semantic methods,^8^ where the core idea of nonsemantic methods is to use the known information in the image to diffuse or fill the area to achieve restoration. The more mainstream nonsemantic methods are based on total generalized variation,^9^ multipyramid-local patch statistics-sparse representation,^10^ fractional-order nonlinear diffusion driven by differential curvature,^11^ domain decomposition type of numerical algorithms,^12^ and other image inpainting algorithms, which have promising results for images with small missing regions or without complex structures and often show blurring for images with larger missing regions or more complex texture information. Employing semantic methods, otherwise known as learning-based image inpainting techniques, deep learning is employed to capture learning of high semantic information concerning the image, comprising of the position, shape, color, and texturing of objects. Through this data distribution, masked domains are filled with regularity. The more mainstream semantic methods can be divided into two types: convolutional neural network (CNN)-based and generative adversarial network (GAN)-based methods.

An analysis conducted not long ago regarding image inpainting via CNN was presented by Zheng et al.^13^, whereby they proposed a form of nonlocal patch-based fully connected tensor network decomposition to apply to multispectral image inpainting. Han et al.^14^ put forth a generative network, with minimal computing power requirements, for image inpainting through enhancement of feature contrast. Schrader et al.^15^ created a groundbreaking neural algorithm that utilised Euler's elastica to reproduce inpainting and applied the concept of deep energy, where the variational energy acts as the neural network loss. In a recent GAN-based image inpainting study, Wang et al.^16^ developed an end-to-end framework termed Automatic Consecutive Context-Perceived Transformer GAN for serial imaging blindly inpainted automatically. Zheng et al.^17^ proposed an encoder equipped with Fourier Convolutional Blocks, allowing it to extract multiscale feature representations from an input image with holes, and a dual-stream decoder featuring a new cascaded global-spatial modulation block at various scale levels for a cascaded modulation GAN. Chen et al.^18^ presented an innovative Multiscale Patch GAN system which implements edge detection image inpainting. The research results of image inpainting in various fields, such as facial recognition, medicine, and natural scenes, show that GAN-based image inpainting has achieved better results than other methods in every field. Due to the relative immaturity of iris image inpainting research and the unique “multidegree of freedom” topology and “high entropy” random texture of the iris, some deep-learning-based iris inpainting methods cannot fully utilize global information, and the generated image still suffers from distortion, noise, blur, and other problems.

Thus, we propose a two-stage deep residual attention-GAN (TSDRA-GAN), with a coarse repair network in the first stage to help the model locate the repair region and coarse texture generation and a fine repair network in the second stage to generate fine iris textures. In the fine repair network, our method uses improved residual blocks as the encoding layer of the model to extract iris texture features more comprehensively and effectively and uses a novel skip connection module to achieve the interfusion of semantic and spatial information of high- and low-level features.

The main contributions of this paper are as follows.

• The first TSDRA-GAN for repairing iris textures obscured by eyelids and eyelashes is proposed in this paper. The two-stage coarse-to-fine generation approach of this method ensures global and local consistency of the generated images and improves the texture distortion and blurring problems of the generated images to a certain extent.
• The method presented in this paper truly achieves texture restoration on the original iris image rather than generating an image consistent with the original image, which is fundamentally different from existing studies on iris image inpainting.
• This paper provides a description of an improved deep residual block for the coding stage of TSDRA-GAN. The deep residual block reuses the middle-layer features based on the normal residual block to avoid the performance degradation caused by the network being too deep.
• In this paper, a new dual-effective attention computation module is presented. This module combines two attention mechanisms to more efficiently fuse the semantic and spatial information of high- and low-level features, avoiding the information loss caused by the maximum pooling operation and the overpassing of low-resolution information.

### Related work

The iris recognition process based on deep learning usually consists of three parts: iris segmentation, normalization, and iris recognition, among which iris segmentation and iris recognition are the two indispensable main tasks,^19^ while normalization of nonconnected low-quality iris images introduces more noniris information and affects recognition accuracy.^20^ Thus, in recent years, some scholars have proposed iris recognition without normalization,^21^^,^^22^ while low-quality iris image inpainting for each type of iris information deficiency has gradually become a hot research topic.

#### Iris inpainting

Depending on the type of iris texture loss, current iris image inpainting algorithms can be broadly classified into edge texture repair and internal texture repair algorithms. Chen et al.^23^ proposed a U-Net optimized discriminator-based GAN for repairing edge-deficient iris textures, which maintains the global consistency of the synthesized images by providing global image feedback. Zeng et al.^24^ proposed the progressive growth of a GAN-based iris image inpainting network for repairing light spot-obscured iris textures that is gradually trained to generate high-resolution images starting from low-resolution images. Zhang et al.^25^ proposed two-way iris patching guided by a region-attention mechanism for repairing light spot-obscured iris textures. Lee et al.^26^ proposed a GAN-based iris image deblurring algorithm for enhancing iris image quality.

#### Residual structure and attention module

The depth of a network model has a key impact on its performance. Theoretically, the deeper the network is, the stronger the feature extraction ability; however, experiments show that when the depth of the network reaches a certain level, the effect of the model approximately saturates or even declines. A network that is too deep may encounter gradient disappearance or gradient explosion. To solve this problem, He et al.^27^ proposed the idea of residual learning, which alleviates the difficulties of training deep neural networks to a large extent. In this paper, we deepen the structure depth of a standard residual block and reuse the middle-layer features of the improved residual block to avoid the performance degradation caused by a network that is too deep.

Li et al.^28^ proposed the introduction of a contextual attention module for improving the global and local semantic consistency of generated images. Cha et al.^29^ proposed a dynamic attention module for reducing the pixel inconsistency problem arising from false textures in generated images. Additionally, Mao et al.^30^ proposed a self-attentive module for extracting the self-similarity of local features of images. Based on the features of iris images, we propose a dual-effective attention computation module combining squeeze and excitation (SE) and spatial attention (SA), where SA can effectively learn the location information of the region to be repaired and SE can effectively learn the feature information of the complete region and use it to repair the missing region. The two blocks enable the module to achieve more efficient extraction and fusion of semantic and spatial information of high- and low-level features and avoid the information loss and excessive transfer of low-resolution information caused by the maximum pooling operation.

### Experimental process and result analysis

#### Experimental environment and parameters

##### Experimental hardware

All experiments in this paper are conducted using the equipment shown in Table S1.

##### Experimental parameters

In the inpainting experiments, the learning rates of the coarse network, fine network, and discriminator are all set to 5e-4, the first-order decay rate is 0.5, the batch size is 8, the number of training epochs is 100, and the ratio of the training weights of the generator and discriminator is 1:5. In the recognition experiment, the learning rate is set to 0.0001, the batch size is 16, and the training epoch is 200.

#### Databases and preprocessing

##### Database

A) The CASIA Iris Image Database V4 Interval (CA4I),^32^ provided by the Institute of Automation of the Chinese Academy of Sciences, contains a total of 2639 images from 249 subjects. The images have clear iris texture information.
B) The IITD iris database^33^ was provided by IIT Delhi, New Delhi, India, and contains a total of 2240 images from 244 subjects. There are 10 images of the left and right eyes of each subject, except for the first 13 subjects, for whom there were only 10 images of the left eye and no images of the right eye. The serial numbers of the other 211 subjects included the first 5 images of the left eye and the last 5 images of the right eye.
C) The ND-IRIS-0405 iris database^34^ consists of 64980 iris images from 356 subjects acquired by an LG 2200 sensor; there were 68 categories of iris images in ND-IRIS-0405 that did not satisfy the requirement of having both 25 left-eye images and 10 right-eye images. Hence, to ensure consistency and fairness in the number of training images per subject in the recognition experiments, the first 25 left-eye and the first 10 right-eye images from the remaining 288 categories, a total of 10,080 images, were assigned to a new subset of ND-IRIS-0405. This subset was used for the inpainting and recognition experiments.

##### Image preprocessing

**Figure 3:**
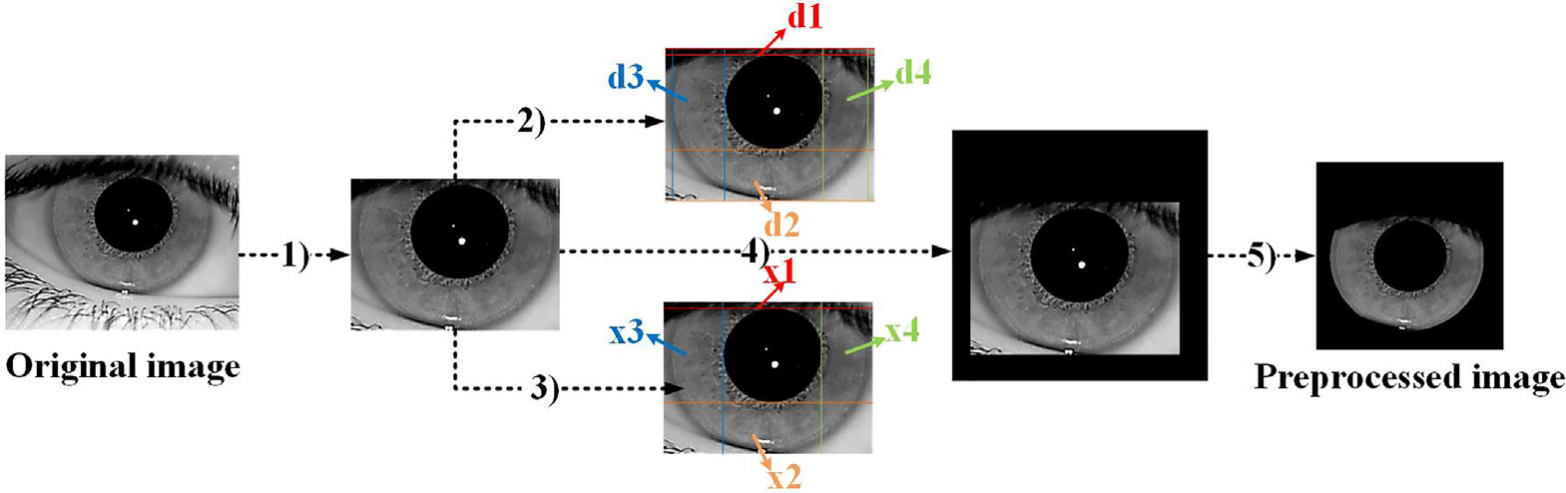
Original iris image preprocessing process

In the inpainting experiments, the results of interpixel logical summation between the original acquired iris images and their corresponding publicly available GT images from the three datasets were used as the original images to be restored. Since the ND-IRIS-0405 dataset does not have a complete publicly available manually annotated GT image, the best segmentation result obtained in recent years for this dataset was chosen as its corresponding GT image.^22^ Since the location of the iris region in the original image is random, i.e., the distance between the outer boundary of the iris region and the image boundary may be too large or too small, the proportion of the iris region in the image may be too large or too small. This is not conducive to the repair of the missing region; thus, in this paper, the original image is preprocessed with the procedure outlined in steps **A)**–**E)** below. The preprocessed original image is recorded as the preprocessed image, and the preprocessing process is shown in Figure 3.

A) Crop the noniris regions in the original acquired iris image and its GT image;
B) Detect the width of the iris area in four directions on the top, bottom, left, and right (denoted as d1, d2, d3, and d4, respectively) of both the original image and GT image;
C) Detect the distance between the inner border of the iris and the image border in the four directions of the top, bottom, left, and right of the original and GT images (denoted as x1, x2, x3, and x4, respectively);
D) Fill the background of size Max (d1, d2, d3, d4) – x + c for each of the four directions above and below the original and GT images (x = x1, x2, x3, x4; c is a constant that is set to 20);
E) Unify the original and GT images and resize the result to 256 × 256.

**Figure 4:**
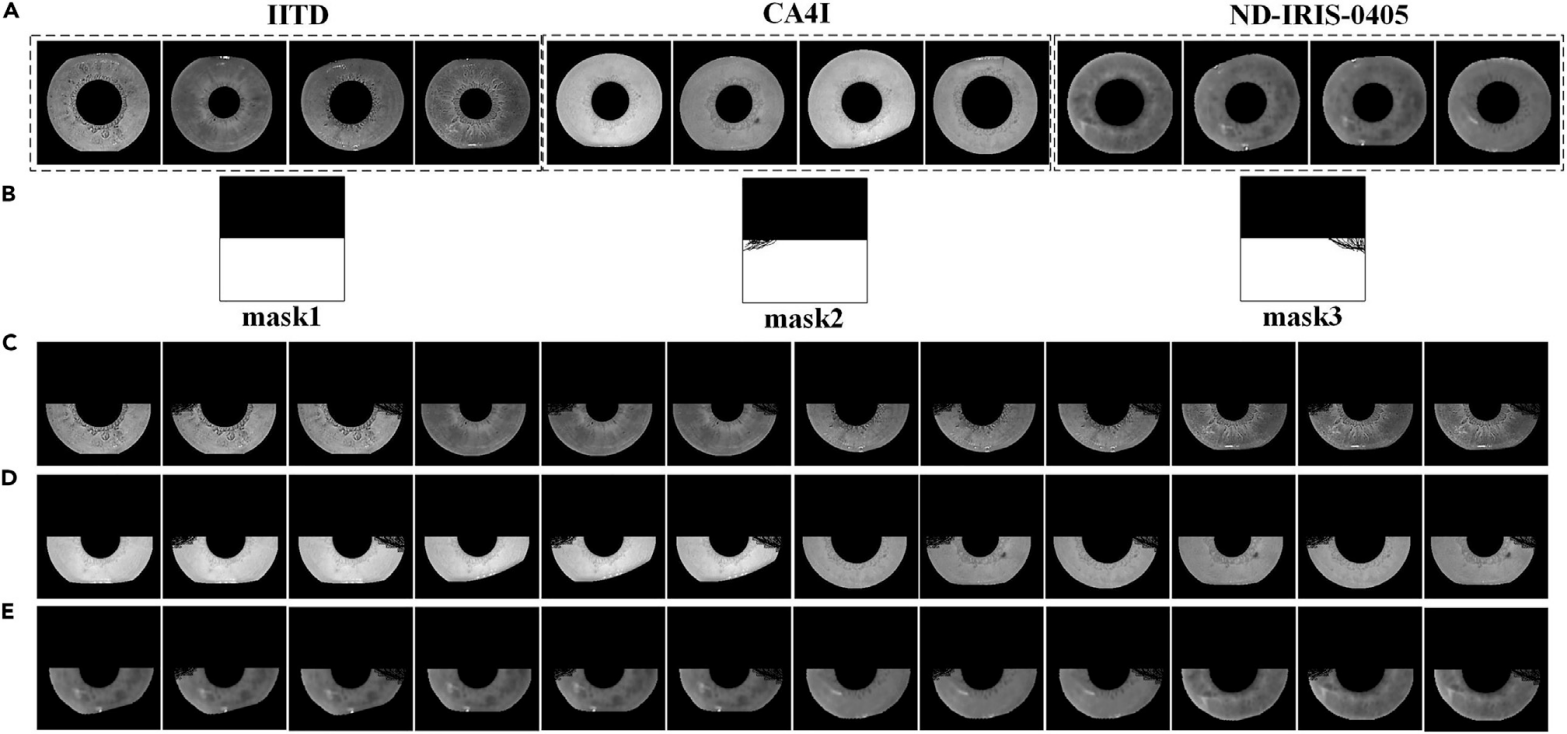
Sample data processing The data samples are shown in Figure 4, where row (A) shows the training control learning group samples of the three datasets, row (B) shows the three mask images, and rows (C), (D), and (E) show the training data after the masks in row (B) have been applied to the images in row (A).

After data preprocessing, the iris images with relatively intact iris regions are selected as the training control learning group in the three datasets. Specifically, 229, 367, and 2480 images were selected from the IITD, CA4I, and ND-IRIS-0405 datasets, respectively. 8-fold data expansion (clockwise and counterclockwise rotation, flip, up, down, left, right, and center crop) was then performed on the iris images selected from the IITD and CA4I datasets, and 2-fold expansion (counterclockwise rotation) was performed on the iris images selected from the ND-IRIS-0405 dataset. At the same time, to simulate the eyelash masking situation in real scenes and to enrich the data diversity to improve the test performance, we apply three kinds of mask images, as shown in Figure 4, by observing the iris image characteristics of the dataset used for the experiment, and the expanded data are logically combined with the three kinds of mask images. Then, the final training data are obtained, in which the IITD, CA4I, and ND-IRIS-0405 datasets contain 6183, 9909,and 14880 samples, respectively.

After completing the model training, all the preprocessed data in the three datasets are input into the model as the test set to obtain the corresponding restored image (painted image). To verify the restoration effectiveness, the investigation presented in this paper entails conducting completely fair and equal recognition experiments on the original images, preprocessed images, and inpainting images, which have completely identical training sets, test sets, training parameters, network structures, etc. To ensure the universality of the conclusions of the recognition experiment, the AlexNet^35^ model is chosen as the recognition network.

#### Result analysis

**Figure 5:**
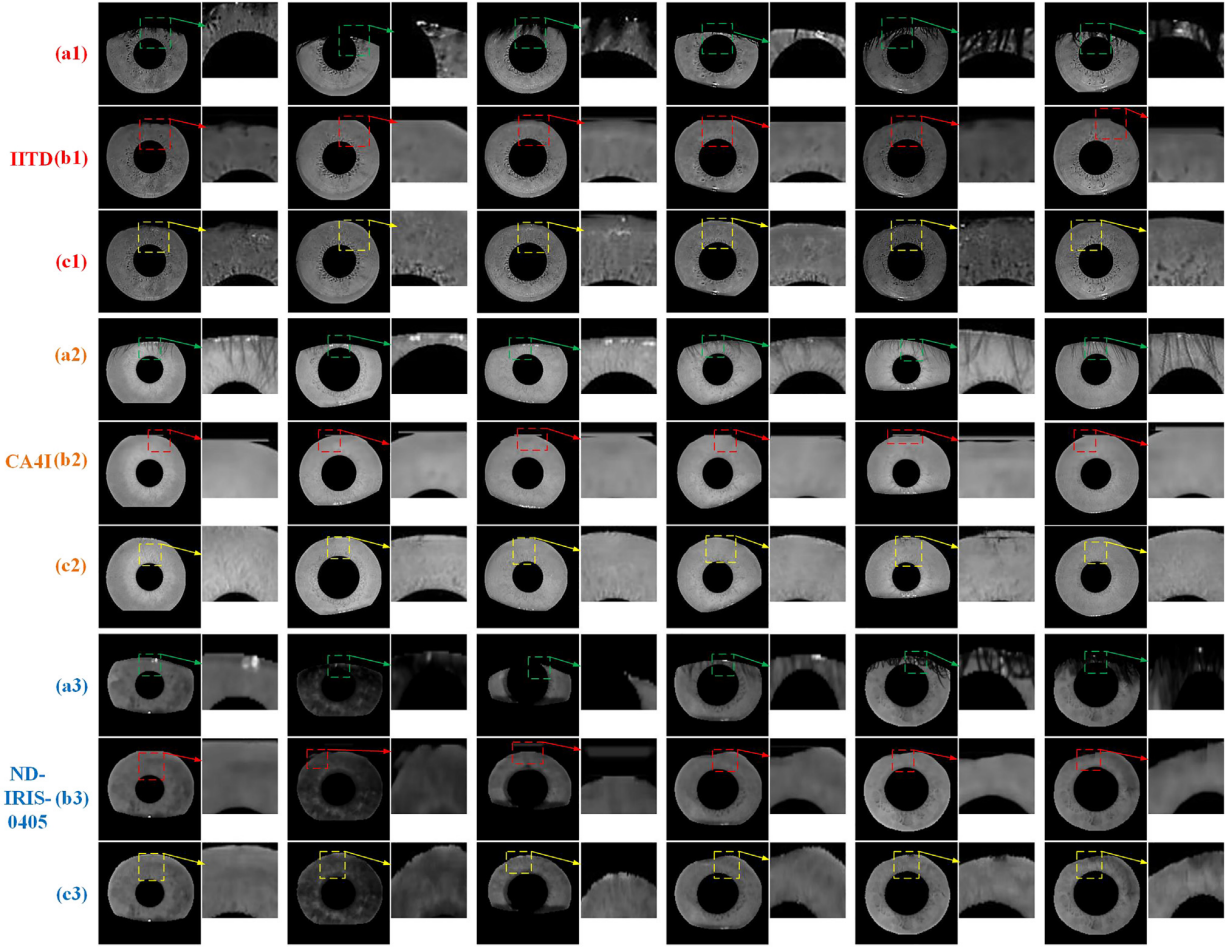
Sample TSDRA-GAN inpainting images

Figure 5 shows the restoration results of TSDRA-GAN for representative samples from the three datasets, where (a1), (a2), and (a3) represent the preprocessed images of the three datasets, (b1), (b2), and (b3) represent the coarse restoration results obtained by the coarse network of TSDRA-GAN for the three datasets, and (c1), (c2), and (c3) represent the fine restoration results, i.e., the inpainting images, obtained by TSDRA-GAN for the three datasets. The images indicated by arrows in the figure are magnifications of local areas in the three types of images to show the coarse-to-fine restoration process more intuitively. The subjective visual analysis shows that the coarse restored images represented by (b1), (b2), and (b3) have repaired image contours and partially repaired textures, but there is texture blurring. The inpainting images represented by (c1), (c2), and (c3) are better restored despite part of the iris texture being obscured by eyelashes and eyelids in all three datasets, and the images have clearer textures and finer contours than the coarse restored images. The iris texture in the restored area is not a fragment of the iris texture present in the intact area, there is no significant color difference between the restored area and the intact area, the restoration marks are very subtle, and the texture distortion is greatly improved. The iris texture in the repaired area is not disorganized but is naturally aligned with the intact iris texture, and the direction of the iris texture conforms to the biological pattern of “radial arrangement near the pupil and circular arrangement around the periphery.”^36^

In addition to the subjective evaluation, the peak signal-to-noise ratio (PSNR) and Fréchet inception distance (FID) are selected as objective evaluation indices for the restoration experiments, and their values are calculated from the preprocessed image and inpainting image. The detailed data are shown in Table S2. The PSNR values of the restored images from the IITD, CA4I, and ND-IRIS-0405 datasets were **22.201, 21.857,** and **24.402**, respectively, and the FID values were **28.229, 16.313,** and **42.635**, respectively.

**Figure 6:**
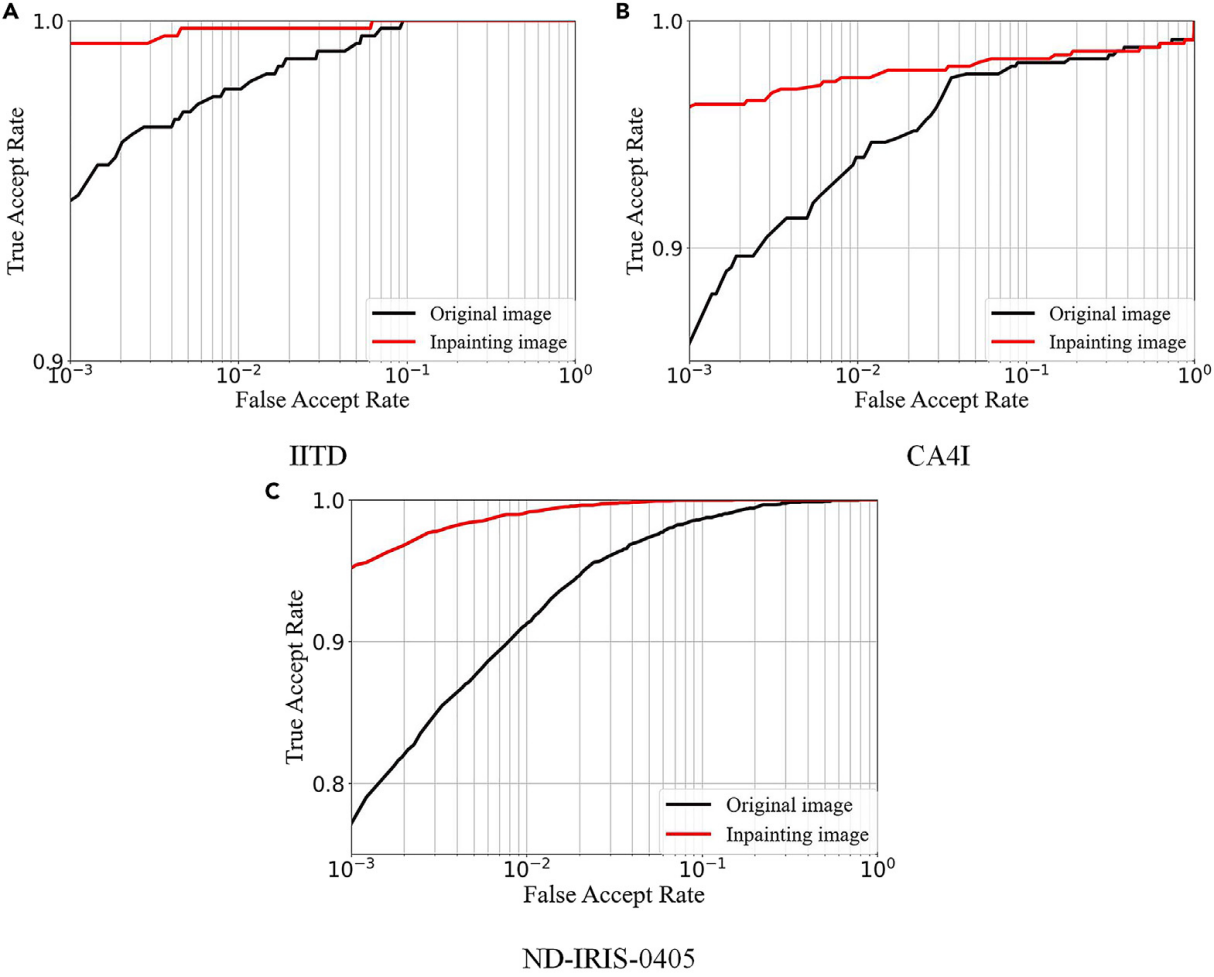
Roc graphs of Original and inpainting images (A) IITD database ROC graphs of the original image and the inpainting image. (B) CA4I database ROC graphs of the original image and the inpainting image. (C) ND-IRIS-0405 database ROC graphs of the original image and the inpainting image.

To confirm the improvement in recognition accuracy for the inpainting image obtained by TSDRA-GAN, the inpainting image and the original image are subjected to identical iris recognition experiments, and the equal error rate (EER) and true accept rate (TAR) at a false accept rate of 0.1% are chosen as the identification evaluation index. The receiver operating characteristic (ROC) curves and recognition metrics of different types of images under the three datasets are shown in Figure 6 and Table S3, respectively.

From Table S3, the EER and TAR of the inpainting image in the three datasets improved significantly compared to those of the original image. The EER of the inpainting image in the IITD, CA4I, and ND-IRIS-0405 datasets reached 0.038%, 1.422%, and 0.561%, respectively. The corresponding TAR values are 99.330%, 96.194%, and 95.174%, respectively. The EER improved by 0.995%, 1.500%, and 2.856%, respectively, and the TAR improved by 4.633%, 10.547%, and 18.072%, respectively, compared to the original image.

#### Ablation experiment

To demonstrate the validity of the structure proposed in this paper and the effect of data preprocessing operations on the recognition accuracy, four sets of ablation experiments are conducted on the IITD, CA4I, and ND-IRIS-0405 datasets. The ablation experiments involve the following procedures:

Ablation 1: To investigate the effect of data preprocessing on recognition accuracy and thus further illustrate the effectiveness of restoration, we conduct recognition experiments on preprocessed images.

Ablation 2: To explore the effectiveness of the dual-attention computing connection (DACC) structure, we replace the DACC with a regular skip connection, i.e., a direct Concat.

Ablation 3: To explore the superior performance of the DACC structure, the DACC is replaced with a convolutional block attention module (CBAM).^37^

Ablation 4: To explore the superior performance of the DACC structure, the DACC is replaced with SA.^37^

The experimental environment, network training parameters, and evaluation metrics of the above three sets of ablation experiments are kept consistent with TSDRA-GAN.

**Figure 7:**
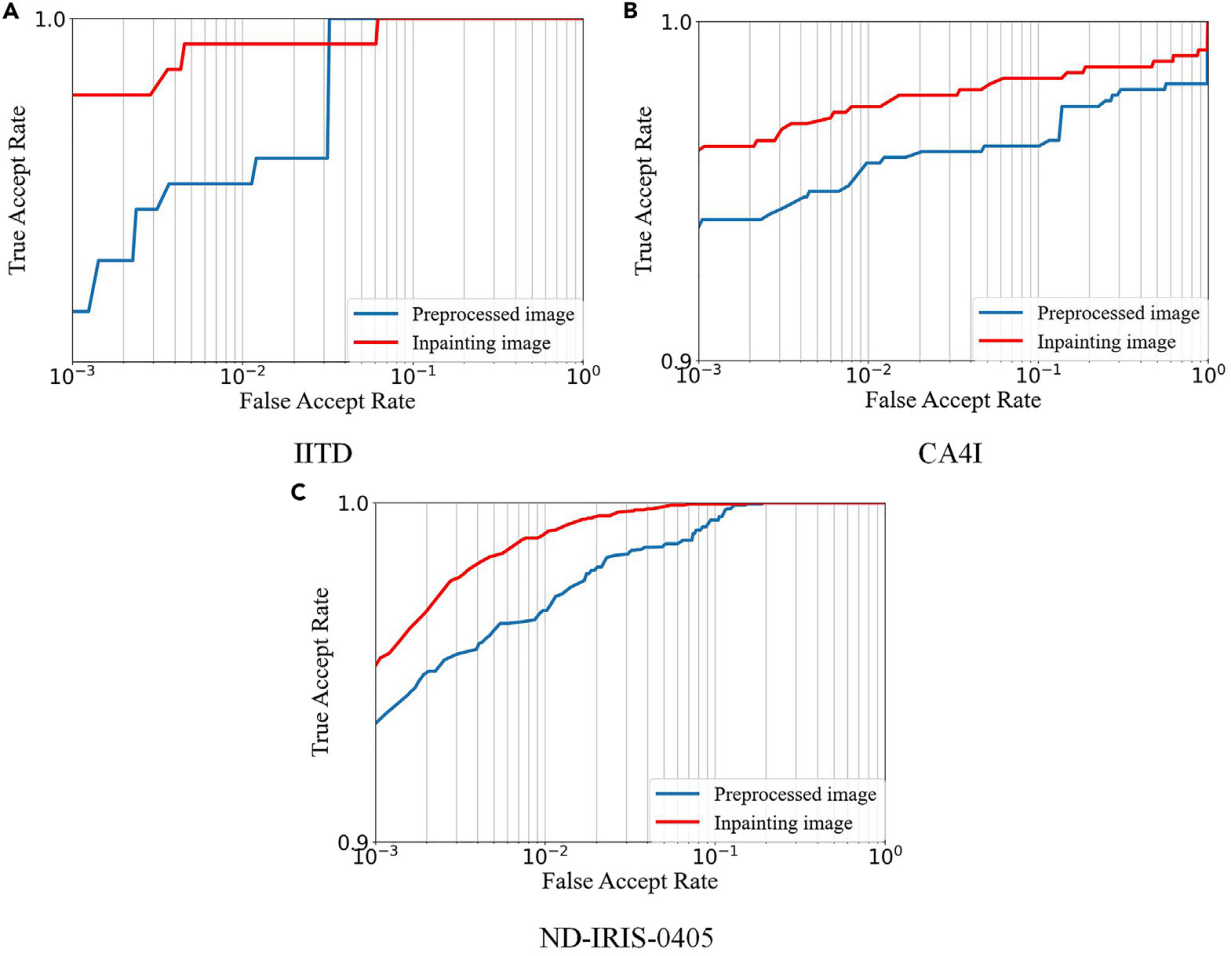
Roc graphs of preprocessed and inpainting images (A) IITD database ROC graph of the preprocessed image and inpainting image. (B) CA4I database ROC graph of the preprocessed image and inpainting image. (C) ND-IRIS-0405 database ROC graph of the preprocessed image and inpainting image.

Figure 7 and Table S4 show the comparative experimental results of Ablation1, containing the ROC curves and EER and TAR values derived from the preprocessed image and inpainting image under fully fair conditions. Preprocessing increases the proportion of the iris region in the whole image, i.e., the foreground proportion increases and the background proportion decreases, which facilitates feature extraction by the network and thus improves the recognition rate. However, in the three datasets, the inpainting image outperforms the preprocessed image by different degrees in each index, and the recognition accuracy for the inpainting image represented by the red curve is higher than that of the preprocessed image represented by the blue curve in the ROC graph. The following two conclusions can be drawn: **(1)** increasing the proportion of foreground in the image can improve the recognition rate to a certain extent; **(2)** the inpainted image obtained by TSDRA-GAN has higher recognition accuracy than the preprocessed image, which confirms the effectiveness of its restoration.

**Figure 8:**
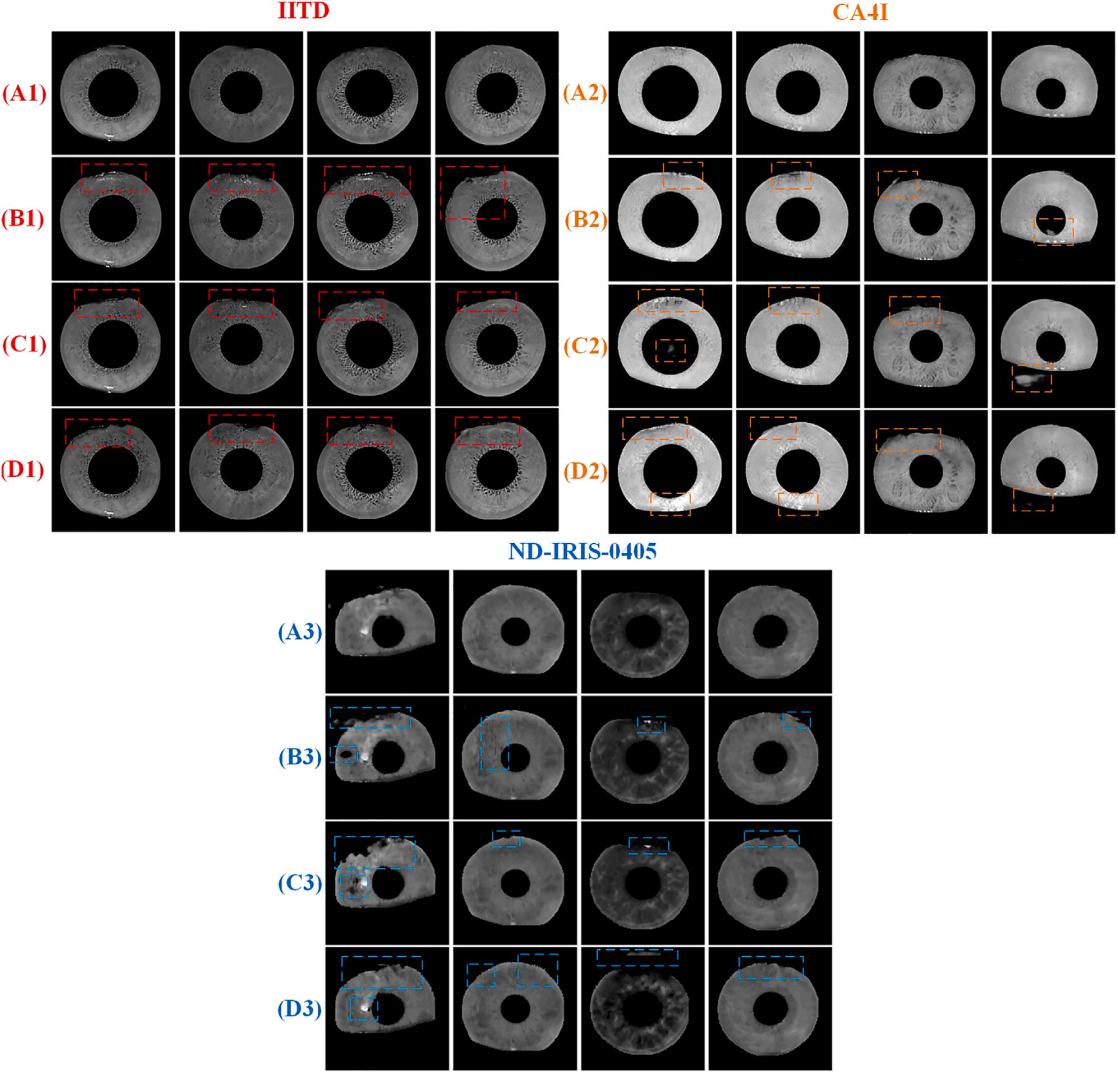
Sample comparison of TSDRA-GAN, Ablation2, Ablation3, and Ablation4 inpainting images

Figure 8 and Table S5 show the inpainting experimental effects of Ablation2, Ablation3, and Ablation4. (A1), (A2), and (A3) in Figure 8 represent the inpainting images obtained by TSDRA-GAN for the three datasets. (B1), (B2), and (B3) represent the Inpainting_sc image obtained by Ablation2 for the three datasets. (C1), (C2), and (C3) represent the Inpainting_cbam image obtained by Ablation3 for the three datasets. (D1), (D2), and (D3) represent the Inpainting_sa image obtained by Ablation4 for the three datasets. As shown in Figure 8, the image quality of the inpainting images obtained by TSDRA-GAN is better than that of the inpainting images obtained by Ablation2, Ablation3, and Ablation4 by different degrees under the same experimental configuration, and the deficiencies of the Inpainting_sc, Inpainting_cbam, and Inpainting_sa images compared with the inpainting image are marked by the dashed boxes in the figure. In the IITD dataset, the Inpainting_sc image represented by (B1) is prone to white spots. The Inpainting_cbam image and Inpainting_sa image represented by (C1) and (D1), respectively, are prone to incomplete repair and edge roughness. In the CA4I dataset, (B2) and (D2) are prone to edge hairiness and overrepair, and (C2) is prone to overrepair. In the ND-IRIS-0405 dataset, (B3), (C3), and (D3) are all prone to incomplete repair and internal hollowing. In terms of the visual effect, the image quality of the inpainted image obtained by TSDRA-GAN is superior.

Table S5 shows the PSNR and FID values of the four types of images. The inpainting images of the three datasets are better than the Inpainting_sc image, Inpainting_cbam image, and Inpainting_sa image in terms of PSNR metrics. The Inpainting_cbam image of the IITD and CA4I datasets is slightly better than the inpainting image, and the inpainting image is better than the Inpainting_sa image in terms of FID metrics. The inpainting image of the ND-IRIS-0405 dataset is better than the other three types of inpainting images. The combined visual effect and inpainting evaluation indices show that the restoration effect of the inpainting image is better than that of the other three types of images.

**Figure 9:**
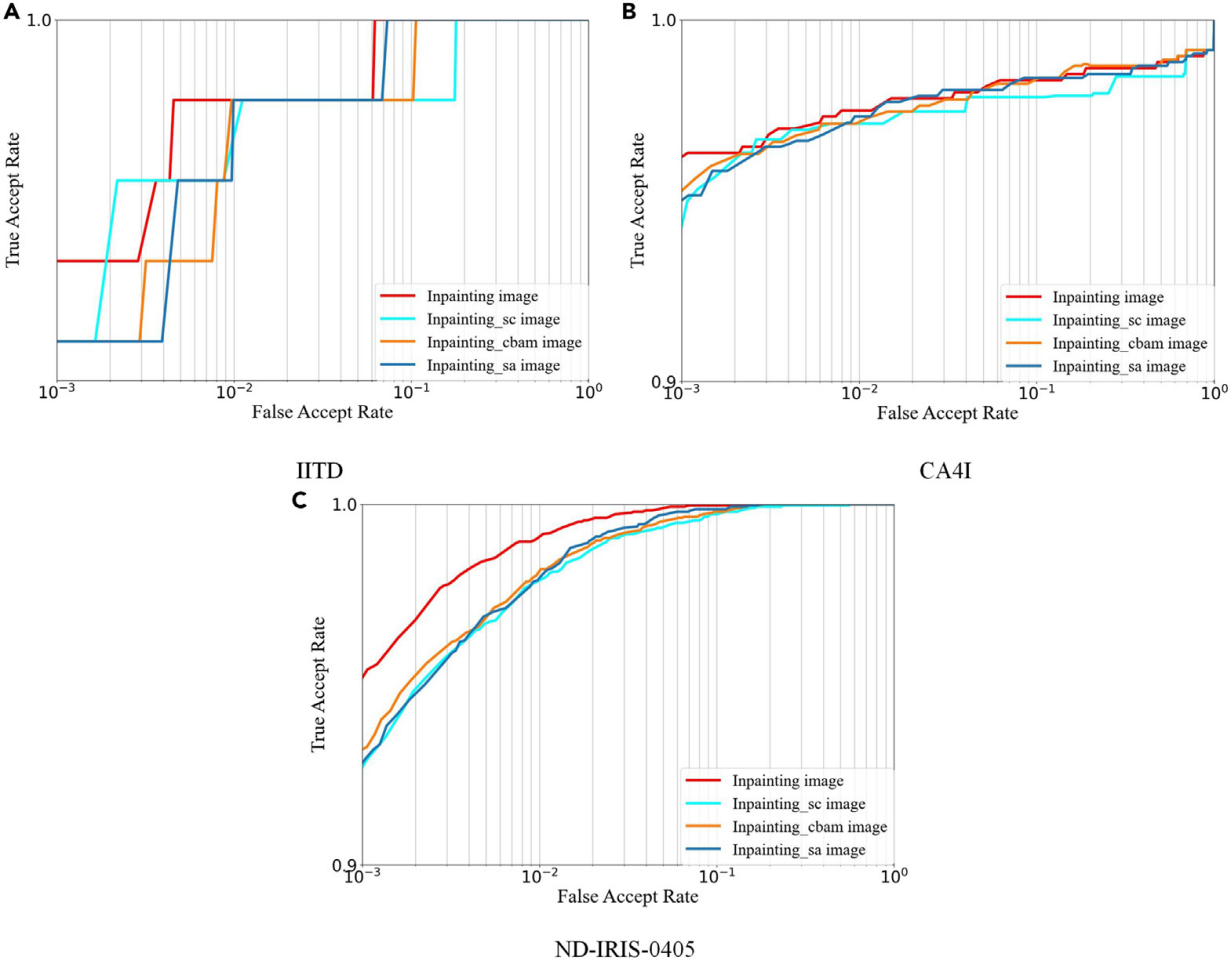
Roc curves of inpainting images (A) IITD database ROC curves of TSDRA-GAN, Ablation2, Ablation3, and Ablation4 inpainting images. (B) CA4I database ROC curves of TSDRA-GAN, Ablation2, Ablation3, and Ablation4 inpainting images. (C) ND-IRIS-0405 database ROC curves of TSDRA-GAN, Ablation2, Ablation3, and Ablation4 inpainting images.

To further illustrate the superior restoration effect of the inpainting images, we conduct fully equivalent recognition experiments on the four types of inpainting images, and Figure 9 and Table S6 show the comparative recognition accuracy for the three types of images. Figure 9 shows that the inpainting image represented by the red line has higher recognition accuracy than the Inpainting_sc image represented by the green line, the Inpainting_cbam image represented by the yellow line, and the Inpainting_sa image represented by the blue line in all three datasets. Meanwhile, from the data in Table S6, the EER and TAR index values of the inpainting image in the three datasets are the best of its kind. As a result, the proposed DACC structure performs better on the iris image inpainting task by combining the inpainting and recognition experiments.

### Conclusions and future work

We propose a two-stage deep residual attention-GAN for repairing eyelid- and eyelash-obscured iris textures called TSDRA-GAN; notably, the objective of TSDRA-GAN is to repair the original iris image rather than artificially create a residual texture on the original iris image. TSDRA-GAN uses a coarse-to-fine generation method, which performs contour repair and coarse repair of the iris texture in the first stage and fine repair of the iris texture in the second stage, and the two-stage generation method ensures the preservation of semantic and texture information of the generated images. In the second stage of the fine network, the residual structure is used as a component of the encoder, and the modified residual block structure further extracts features and mitigates the performance degradation caused by the deepening of the network. In addition, in the skip connection part of this phase, DACC performs bidirectional computational fusion of encoder and decoder features, and it performs relevant computations to filter out important features according to their different characteristics, achieving more effective information fusion for iris repair tasks. The proposed restoration scheme is found to be feasible and effective through extensive qualitative and quantitative inpainting and recognition experiments conducted on three publicly available iris datasets.

For iris image inpainting tasks, there are various forms of missing iris textures, and the current mainstream supervised learning approach, although better at generating quality images, requires the presence of learning objects. It is difficult to produce control learning groups for each missing texture in realistic scenarios. Thus, using an unsupervised learning approach for iris texture repair and reducing human involvement in the process will be a major research direction in iris image inpainting in the future.

## Declaration of AI and AI-assisted technologies in the writing process

During the preparation of this work the author(s) used chatGPT in order to enhance the English grammar and paraphrase some sentences. After using this tool/service, the author(s) reviewed and edited the content as needed and take(s) full responsibility for the content of the publication.

## STAR★Methods

### Key resources table

**Table:** 

REAGENT or RESOURCE	SOURCE	IDENTIFIER
**Deposited data**		

CASIA Iris Image Database V4 Interval	Chinese Academy of Science—Institute of Automation	http://biometrics.idealtest.org/dbDetailForUser.do?id=4. 2020-01-15
IITD iris database	IIT Delhi, New Delhi, India	https://doi.org/10.1016/j.patcog.2009.08.016
ND-IRIS-0405 iris database	Bowyer et al.^34^	https://doi.org/10.48550/arXiv.1606.04853

**Experimental models**		

TSDRA-GAN	This paper	N/A
Alexnet	Krizhevsky et al.^35^	https://dl.acm.org/doi/10.1145/3065386

**Software and algorithms**		

Python3.6	Python Software Foundation	https://www.python.org
Tensorflow	google	www.tensorflow.org

### Resource availability

#### Lead contact

Further information and requests for related data should be directed to and will be fulfilled by the lead contact, Yudong Zhang (yudongzhang@ieee.org).

#### Materials availability

This study did not generate new materials.

### Method details

The proposed approach consists of coarse network, fine network and discriminator, modified residual block, dual attention computing connection, explained in detail in this section.

In the field of iris image inpainting, due to the high coherence of iris textures and their unique "high entropy rate", the common shortcomings of existing iris image inpainting algorithms are distortion, blurriness, and obvious restoration traces in the generated images. In iris image inpainting, the goal is to restore the defective image to match the original iris image; however, many current methods artificially mask the defective images used in the experiments on top of the original iris image, which is not a true restoration of the original iris image. In addition, eyelash and eyelid occlusion is one of the most common types of missing iris textures; therefore, this paper presents TSDRA-GAN for repairing iris textures with eyelash and eyelid occlusion. The detailed structure of the proposed network is shown in Figure 2.

TSDRA-GAN consists of a coarse network, a fine network, and a discriminator, where the coarse network is responsible for the coarse repair of iris texture and performs contour repair and coarse repair of iris texture on the original image. The network in this stage calculates the L1 loss with the ground truth (GT), repairs the contour of the area to be repaired and generates the coarse iris texture. Then, the generated image is used as the input of the fine network instead of the original image. The fine network performs texture refinement restoration on the input, and the generated image in this stage is input to the discriminator together with GT to calculate the WGAN-GP loss. In calculating the slopes in the WGAN-GP gradient penalty formula, a smoothing constant C (C=1e-8) is introduced in this paper to effectively avoid the NaN value problem. The two-stage training approach enables a smooth transition from semantic information to texture details in the image generation process, which ensures the local and global consistency of the generated images and alleviates the texture distortion of the restored iris images to a certain extent.

#### Coarse network

The coarse network consists of an encoder, transition layer, decoder and regular skip connection, where the encoder has four layers, each containing two Conv3×3-BN-LReLU modules composed of a 3×3 convolution, batch normalization (BN) and leaky rectified linear unit (LReLU), and a maximum pooling layer. The transition layer is located after the encoder, which is used for further extraction of the encoder output features and as the input of the first layer of the decoder, and consists of two Conv3×3-BN-LReLU modules. The decoder also contains four layers; each layer contains an upsampling operation, a Concat operation and two Conv3×3-BN-LReLU modules, where the upsampling operation is implemented using nearest neighbor interpolation. Finally, the output of the last layer of the decoder is the image channel limited using a 1×1 convolution to obtain a coarse restored image of the same size and channel as the original image. The generated image at this stage is directly input to the fine network without any manual processing in between.

#### Fine network and discriminator

The fine network consists of the encoder, transition layer, decoder and dual attention computing connection (DACC). The encoder has five layers, the first of which consists of a Conv3×3-BN-LReLU module and maximum pooling layer, which performs coarse extraction of features and removes redundant information from the input image. The next four layers consist of the modified residual block (MRB) and a maximum pooling layer. The modified depth residual block adopts a deeper structure than the standard residual block and reuses the features of the intermediate layers to better extract features and prevent network performance degradation. The transition layer has the same structure and role as the transition layer in the coarse network. The decoder also contains five layers. Each layer contains an upsampling operation and a Conv3×3-BN-LReLU module, where the upsampling operation is implemented by Conv1×1 combined with nearest neighbor interpolation, effectively avoiding the "checkerboard effect" of the generated image and increasing the nonlinearity of the network. Finally, a 1×1 convolution is used for the output of the last layer of the decoder for image channel restriction to obtain a finely restored image of the same size and channel as the original image. DACC is used to fuse semantic and spatial information of high- and low-level features, and it contains two inputs and one output, whose inputs are the output of encoder layer i and the output sampled on decoder layer n-i+1 (n is the number of decoder layers, i∈[1,n]), and whose output is the input of the Conv3×3-BN-LReLU module in decoder layer n-i+1.

The discriminator aims to correctly determine whether the input image is a real image or a generated image and finally achieve Nash equilibrium. Each of the first 6 layers undergoes a convolution operation with a step size of 2 and a convolution kernel size of 5×5 for feature extraction and is then activated by the LReLU function. Due to the introduction of the WGAN-GP training method in the training process, the discriminator does not use the BN operation. The last layer uses Conv3×3-LReLU- Average_Pooling with a step size of 1 to limit the width and height of the feature map to 1, and the input image is classified using a linear fully connected layer. To enable healthy competition between generators and discriminators, we use He initialization in each forward pass process.^31^ Meanwhile, the design of the loss function has a key influence on the stability of the network training and the quality of the generated images, so the method presented in this paper uses the WGAN-GP and L1 losses, where L1 loss is used in the coarse repair phase and WGAN-GP is used in the fine repair phase.

#### Modified residual block

Residual learning helps to improve restoration performance, and the method of stacking multiple residual blocks to design generators is often used in image generation techniques based on GANs. However, increasing the depth of generators consistently leads to an excessive gap between their volume and that of discriminators and increases the risk of gradient explosion or gradient disappearance, exacerbating the difficulty of training GANs. For the generator, it is most important to learn the high-level features (latent features) of the image and disperse them into different low-level features (images) efficiently. In this paper, we extend the standard residual block concept and use two sets of Conv1×1-BN-LReLU and Conv3×3-BN-LReLU modules to further deepen the residual structure, where Conv1×1 is used to change the dimensionality of the feature map so that the number of filters in Conv3×3 is not affected. To avoid problems such as performance degradation caused by deepening the network, the method presented in this paper introduces a new shortcut path between the first and second Conv3×3-BN-LReLU modules to enable the residual structure to internally skip the layers that may lead to performance degradation, pass the original features to the deeper layers and then concatenate the original features with the deeper features to improve the feature reuse rate. The detailed structure of the MRB is shown in Figure 2, and the computational flow is shown in Equations 1, 2, and 3.

(Equation 1) $$y_{1}=W\left(W\left(X_{Input},X_{1}\right),X_{2}\right)$$

(Equation 2) $$y_{2}=W\left(W\left(y_{1},X_{2}\right),X_{3}\right)$$

(Equation 3) $$y_{3}=Add\left(Concat\left(y_{1},y_{2}\right),X_{Input}\right)$$

where $X_{Input}$ is the input of the MRB, $X_{1}$ is the output of the first Conv1×1-BN-LReLU module, $X_{2}$ is the output of the first Conv3×3-BN-LReLU module, $X_{3}$ is the output of the second Conv1×1-BN-LReLU module, $y_{3}$ is the output of the MRB, $y_{1}$ is the first set of Conv1×1-BN- LReLU, Conv3×3-BN-LReLU outputs, $y_{2}$ is the first set of Conv1×1-BN-LReLU, Conv3×3-BN-LReLU outputs, *Add* is the feature map sum operation, *Concat* is the feature map merge operation, and *W()* is the operator relationship between the weights and the inputs.

#### Dual attention computing connection

For iris image inpainting, there are two key tasks: first, the network must effectively learn the location information of the region to be repaired; second, the network must effectively learn the feature information of the complete region and use it to repair the missing region. Based on these tasks, SE and SA are selected as the base structure of the DACC module in this paper. DACC acts as a bridge between the encoder and decoder in the fine network and is used to concatenate the features extracted in the encoder and the features recovered in the decoder to avoid information loss. In contrast to the regular skip connection, which does not filter the past features and to a certain extent leads to information redundancy in the features after Concat, DACC receives both potential features in the encoder and reconstructed features in the decoder and performs relevant calculations to filter out features that have different characteristics. The analysis shows that the features in the encoder are spatially richer due to the pooling layer operation, while the features in the decoder are richer in terms of channel because they are reconstructed from potential features.

As a result, DACC computes the spatial attention value of the feature map in the encoder via SA. However, our SA differs from the standard SA in that our SA retains the post sigmoid value (denoted as $X_{sa}$) without multiplying it with the input features. In addition, the correlation between feature channels in the encoder is strengthened by SE (denoted as $X_{se}$), and then the BN-LReLU operation is performed on the features of the decoder layer, and the result is multiplied by $X_{sa}$. Next, the BN-Sigmoid operation is performed, and the result is multiplied by $X_{se}$. Then, the output of DACC is obtained by comparing the two results and used as the input of the Conv3×3-BN-LReLU module in the current layer of the decoder. The detailed structure of DACC is shown in Figure 2, and the calculation flow is shown in Equations 4, 5, and 6.

(Equation 4) $$X_{se}=\sigma \left(L\left(GAP\left(X_{en}\right),W1\right)W2\right)$$

(Equation 5) $$X_{sa}=\sigma \left({Conv}^{7\times 7}\left(Concat\left(GAP\left(X_{en}\right),GMP\left(X_{en}\right)\right)\right)\right)$$

(Equation 6) $$X_{out}=Concat\left(L\left(BN\left(X_{de}*X_{sa}\right)\right),X_{se}*\sigma \left(BN\left(X_{de}\right)\right)\right)$$

where $X_{se}$ is the encoder layer feature map that enhances the relationship between channels, $X_{en}$ is the output of the encoder layer corresponding to the current DACC layer, $X_{sa}$ is the spatial attention value of the encoder layer feature map, $X_{de}$ is the output of the decoder layer corresponding to the current DACC layer, $X_{out}$ is the DACC output, σ is the sigmoid operation, *L* is the leaky ReLU operation, *BN* is the batch normalization operation, *GAP()* is the global average pooling operation, *GMP()* is the global maximum pooling operation, *Concat* is the feature map merge operation, ∗ is the feature map multiplication operation, and *W1* and *W2* are the weights of the two fully connected layers.

### Quantification and statistical analysis

Detailed description of iris image inpainting methods is provided in experimental process and result analysis under the following sections: experimental environment and parameters; databases and preprocessing; result analysis; ablation experiment. The overall experiments are conducted in the same hardware and PyCharm 2022.2.3 software environment. In the image inpainting problem, larger PSNR metric values and smaller FID metric values represent higher quality inpainting images. In recognition experiments, smaller EER and larger TAR represent higher image recognition accuracy. Figures 3 and 4 show the raw image pre-processing process and the results. Tables S2 and S3, Figures 5 and 6 show the restoration results and recognition accuracy of the inpainting images. Tables S4, S5, and S6, Figures 7, 8, and 9 show the experimental results of a series of ablation experiments, which demonstrate the effectiveness of the method in this paper.

## Data Availability

The dataset that informed or guided this study are available online and data reported in this paper will be shared by the lead contact upon request. A link to code and DOIs are listed in the key resources table.• All data reported in this paper will be shared by the lead contact upon request. • The original code is not reported in this paper. • Any additional information required to reanalyze the data reported in this paper is available from the lead contact upon request.

## Supplemental information

Figure S1. Overall structure for repair iris textures obsured by eyelids and eyelashes, related to STAR Methods
