# Supplementary material for: From coarse to fine: Two-stage deep residual attention generative adversarial network for repair of iris textures obscured by eyelids and eyelashes
Source: iScience. 2023 Jun 21;26(7):107169. doi: 10.1016/j.isci.2023.107169 (PMC10359935; doi:10.1016/j.isci.2023.107169)
Supplement: Figure S1. Overall structure for repair iris textures obsured by eyelids and eyelashes, related to STAR Methods [file mmc1.pdf]

**iScience, Volume *26***

## Supplemental information

# From coarse to fine: Two-stage deep residual attention generative adversarial network for repair of iris textures obscured by eyelids and eyelashes

**Ying Chen, Yugang Zeng, Liang Xu, Shubin Guo, Ali Asghar Heidari, Huiling Chen, and Yudong Zhang**

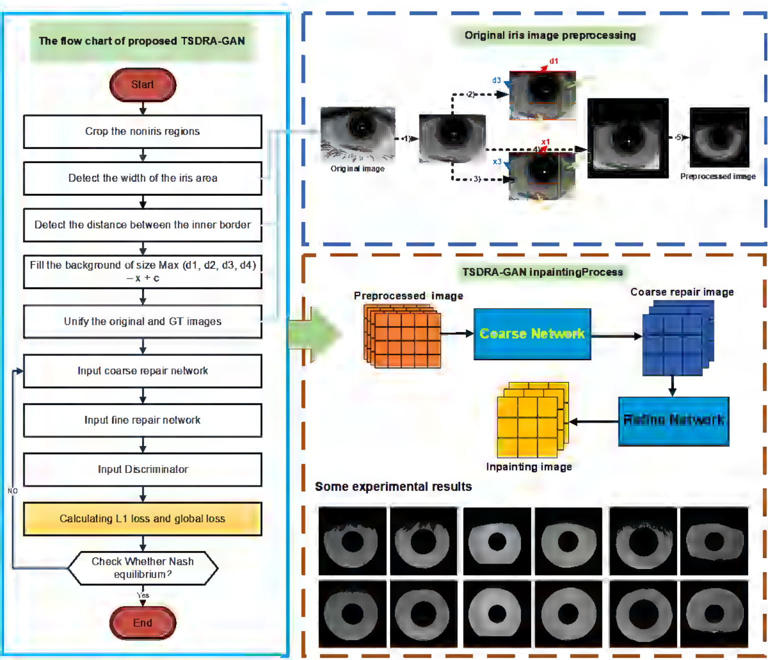
The flow chart of proposed TSDRA-GAN
Start
Crop the noniris regions
Detect the width of the iris area
Detect the distance between the inner border
Fill the background of size Max (d1, d2, d3, d4) – x + c
Unify the original and GT images
Input coarse repair network
Input fine repair network
Input Discriminator
Calculating L1 loss and global loss
Check Whether Nash equilibrium?
NO
Yes
End
Original iris image preprocessing
Original image
d1
d3
x1
x3
Preprocessed image
TSDRA-GAN inpaintingProcess
Preprocessed image
Coarse Network
Coarse repair image
Refine Network
Inpainting image
Some experimental results
