# Supplementary material for: From coarse to fine: Two-stage deep residual attention generative adversarial network for repair of iris textures obscured by eyelids and eyelashes
Source: iScience. 2023 Jun 21;26(7):107169. doi: 10.1016/j.isci.2023.107169 (PMC10359935; doi:10.1016/j.isci.2023.107169)
Supplement: Document S1. Tables S1–S6 [file mmc2.pdf]

Table 1. Experimental equipment parameter settings

Parameter	Details
CPU	Inter(R) Xeon(R) Gold 5118 CPU @ 2.30 GHz
GPU	NVIDIA GeForce RTX 2080Ti
Memory	64.0 GB
Programming Language	Python
Operating System	Windows 10
Neural Network Framework	TensorFlow

Table 2. PSNR and FID values of TSDRA-GAN inpainting images

Dataset	PSNR	FID
IITD	22.201	28.229
CA4I	21.857	16.313
ND-IRIS-0405	24.402	42.635

Table 3. EER and TAR values of the original image and inpainting image

		IITD	CA4I	ND-IRIS-0405
EER	Original image	1.033%	2.922%	3.417%
	Inpainting image	**0.038%**	**1.422%**	**0.561%**
TAR	Original image	94.697%	85.647%	77.102%
	Inpainting image	**99.330%**	**96.194%**	**95.174%**

Table 4. EER and TAR values of the preprocessed image and inpainting image

		IITD	CA4I	ND-IRIS-0405
EER	Preprocessed image	0.143%	1.7420%	0.674%
	Inpainting image	**0.038%**	**1.422%**	**0.561%**
TAR	Preprocessed image	97.437%	93.912%	93.465%
	Inpainting image	**99.330%**	**96.194%**	**95.174%**

Table 5. PSNR and FID values of TSDRA-GAN, Ablation2, Ablation3 and Ablation4 inpainting images

		IITD	CA4I	ND-IRIS-0405
PSNR	Inpainting image	**22.201**	**21.857**	**24.402**
	Inpainting_sc image	21.742	21.224	23.915
	Inpainting_cbam image	22.184	21.586	24.213
	Inpainting_sa image	22.053	21.438	24.006
FID	Inpainting image	28.229	16.313	**42.635**
	Inpainting_sc image	28.052	16.118	43.158
	Inpainting_cbam image	**27.976**	**15.383**	42.896
	Inpainting_sa image	28.317	16.441	43.216

Table 6. EER and TAR values of TSDRA-GAN, Ablation2, Ablation3 and Ablation4 inpainting images

		**IITD**	**CA4I**	**ND-IRIS-0405**
EER	Inpainting image	**0.038%**	**1.422%**	**0.561%**
	Inpainting_sc image	0.054%	1.451%	1.096%
	Inpainting_cbam image	0.040%	1.448%	1.019%
	Inpainting_sa image	0.048%	1.450%	1.057%
TAR	Inpainting image	**99.330%**	**96.194%**	**95.174%**
	Inpainting_sc image	99.107%	94.245%	92.638%
	Inpainting_cbam image	99.107%	95.254%	93.196%
	Inpainting_sa image	99.107%	94.995%	92.813%
